# Co-Design of BW-Enhanced Dual-Path Driver and Segmented Microring Modulator for Energy Efficient Si-Photonic Transmitters

**DOI:** 10.3390/mi17030370

**Published:** 2026-03-19

**Authors:** Yingjie Ma, Bolun Cui, Guike Li, Jian Liu, Nanjian Wu, Nan Qi, Liyuan Liu

**Affiliations:** 1 The State Key Laboratory of Semiconductor Physics and Chip Technologies, Institute of Semiconductors, Chinese Academy of Sciences, Beijing 100083, Chinaliuly@semi.ac.cn (L.L.); 2 The Center of Materials Science and Optoelectronics Engineering, University of Chinese Academy of Sciences, Beijing 100049, China

**Keywords:** dual-segment microring modulator, silicon photonic transmitter, feedforward driver, bandwidth extension

## Abstract

Artificial intelligence computing systems increasingly demand high-bandwidth, high-extinction-ratio, chip-to-chip optical transceivers. Silicon microring modulators (MRMs) are attractive for such transmitters due to their compact footprint and wavelength-division multiplexing capability. However, for a specified extinction ratio, the optical bandwidth for high-Q MRMs and the driver’s RC time constant prevent conventional single-segment MRM drivers from supporting 100 GBaud class PAM4 transmission. This work presents a broadband driver exploiting the feedforward technique for dual-segment MRMs. It extends electro-optical bandwidth while maintaining a high Q-factor and extinction ratio. The input signal is split into low- and high-frequency components that drive the long and short segments of the MRM, respectively. The long segment uses a broadband low-pass driver, whereas the short segment employs a driver with a programmable bandpass response near the Nyquist frequency. The design space is obtained from an equivalent electro-optical model under constant group-delay constraints. Simulations at 1310 nm show that the 3 dB electro-optical bandwidth improves from ~50 to >70 GHz and that a 200 Gb/s PAM4 optical eye diagram exhibits an open eye; the energy efficiency is 1.44 pJ/bit, and the extinction ratio improves from 2 dB to 4.1 dB. The proposed technique provides a tunable electro-optical co-design approach for high-bandwidth-density, high-extinction-ratio silicon photonic transmitters.

## 1. Introduction

With the rapid development of artificial intelligence (AI) and machine learning, the data throughput of computing clusters and data centers is growing exponentially [1]. Traditional electrical interconnections suffer from conductor loss, crosstalk, and I/O density limitations at high frequencies [2,3]. As a result, it is difficult to support chip-to-chip and rack-to-rack data rates at the Tb/s level. Silicon photonics (SiPho), with its high compatibility with CMOS processes and suitability for large-scale integration, has therefore become a primary solution for high-density chip-to-chip interconnects [4]. Among various SiPho devices, silicon microring modulators (MRMs) are particularly attractive for realizing high-bandwidth-density optical transmitters [5]. Silicon microring modulators (MRMs) offer an extremely small footprint, inherent support for wavelength-division multiplexing (WDM), and low drive energy, enabling high-channel-count parallel integration [5]. In high-speed optical IO, MRM-based transmitters must provide a sufficient extinction ratio at symbol rates of 100 GBaud and above. Meanwhile, they must cope with the parasitic capacitance and inductance originating from the modulator itself and from the package. The total parasitics of the driver, package, and microring, together with the limited electro-optical conversion bandwidth, have become key bottlenecks that constrain further bandwidth scaling for such systems [6,7].

Prior work has explored both circuit-level and photonic-level approaches. In the electrical domain, high-speed DACs with multi-tap feedforward equalization in the transmitter (TX FFE) pre-emphasizes the input signal to compensate for the limited electro-optical bandwidth, but they incur high power consumption and considerable design complexity [8]. Inductive-peaking techniques extend analog bandwidth by co-designing on-chip inductors with packaging parasitics. However, large inductors consume area and reduce integration density [9]. They are also sensitive to process and layout variations, which limits their use in high speed transmitters. In the optical domain, segmented MRMs are used as optical DACs for PAM4 signals, and cascaded or segmented rings could be configured as optical feedforward equalizers [10,11]. These structures can relieve the burden on electronic DACs or drivers, but they are highly sensitive to fabrication and temperature variations, introduce insertion losses, and complicate resonance control. Conventional solutions often trade microring Q-factor and modulation efficiency for bandwidth, or trade power and complexity for performance, which limits further improvements in bandwidth density and energy efficiency.

This work proposes a dual-path feedforward driver architecture for dual-segment MRMs. In the electrical domain, the input signal is split into low- and high-frequency spectral components, which are routed to a moderate-bandwidth low-frequency path and a narrowband high-frequency path, respectively. The two band-limited drive signals are applied to the long and short segments of the same dual-segment MRM, such that their modulation contributions combine on a single resonant mode. This effectively extends the overall electro-optical bandwidth while preserving a relatively high Q-factor and extinction ratio for the MRM. The proposed architecture leverages spectral partitioning in the electrical domain and response superposition in the optical domain to alleviate the bandwidth bottleneck with good energy efficiency.

## 2. Feedforward Dual-Segment Microring Driving Technique

### 2.1. Bandwidth–Extinction-Ratio Constraint for a Single-Segment Microring Modulator

An MRM is intrinsically a resonant device. For given coupling and loss conditions, the cavity quality factor Q is essentially fixed. The modulation signal changes the effective index of the ring waveguide and thus perturbs the resonance, which leads to an intensity modulation at the output. For a single-segment MRM, the transmission spectrum can be approximated by a Lorentzian response,

(1) $$H_{opt}\left(f\right)\approx \frac{1}{1+j\frac{f\;-\;f_{0}}{\Delta f_{opt}/2}}$$

where the optical 3 dB bandwidth $\Delta f_{opt}$ is

(2) $$\Delta f_{opt}=\frac{f_{0}}{Q}$$

and $f_{0}$ is the resonance frequency [12,13].

When a depletion-type p–n junction is used to tune the resonance, and the modulation rate approaches or exceeds $\Delta f_{opt}$, the optical field in the ring cannot build up and decay fast enough. The electro-optical response exhibits pronounced high-frequency roll-off and group-delay variation, which manifests as an insufficient electro-optical bandwidth. In addition, bond wires, pads, RDL traces, and other packaging structures introduce extra parasitic capacitance and inductance that further reduce the bandwidth. To achieve a high extinction ratio in the O band, the MRM often requires a high Q. However, a high Q corresponds to a narrow optical bandwidth. With the electrical driver’s response and packaging parasitics, the overall electro-optical 3 dB bandwidth is limited.

### 2.2. Feedforward Dual-Segment Microring Architecture

To alleviate the fundamental trade-off between high extinction ratio (ER) and wide electro-optical (EO) bandwidth in high-Q microring modulators (MRMs), this work combines a dual-segment MRM with a dual-path feedforward driver. The high-speed input is broadcast to two analog driver paths in parallel, and the effective low-/high-frequency separation is realized by the complementary frequency responses of the two paths rather than by explicit switching. The two path outputs drive two electrically isolated modulation segments on the same ring: a long segment (MSB) and a short segment (LSB). Since both segments share the same resonant mode, their modulation contributions are superposed on the same Lorentzian resonance and therefore jointly shape the overall EO transfer function. By allocating the high-frequency correction through the short segment (HF path) while keeping the long segment as the main modulation contributor (LF path), the proposed co-design extends the overall EO 3 dB bandwidth without sacrificing the resonator Q and ER.

**Figure 1:**
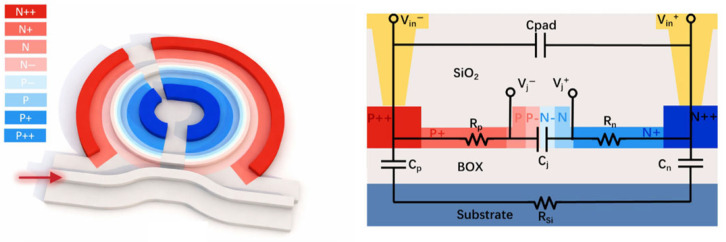
Structure and equivalent circuit model of the two-segment microring modulator [14].

The two-segment silicon microring modulator (MRM) used in this work, as shown in Figure 1, follows the O-band dual-segment depletion-type MRM reported in [14,15]. It is implemented on a standard 220 nm SOI platform and uses a rib waveguide in the modulation region (70 nm slab height, 380 nm width). A lateral PN junction with gradient doping is adopted: the lightly doped junction core uses N−/P− = 3.8 × 10^17^ cm^−3^/2.0 × 10^17^ cm^−3^ with a 50 nm doping offset, supported by N/P = 1.5 × 10^18^ cm^−3^/9.7 × 10^17^ cm^−3^, N+/P+ = 1.0 × 10^19^ cm^−3^/5.3 × 10^18^ cm^−3^, and N++/P++ = 1.1 × 10^20^ cm^−3^/7.1 × 10^19^ cm^−3^; the heavily doped regions are placed about 700 nm away from the waveguide to balance RC loss and optical absorption. The active region along the ring is partitioned into MSB/LSB segments with an approximately 2:1 length ratio, targeting operation near 1310 nm. The ring radius is 6 um, corresponding to an FSR of about 11 nm, and a racetrack coupler (200 nm gap, 1 um coupling length) yields a slightly over-coupled resonance with a measured Q of about 3700.

**Figure 2:**
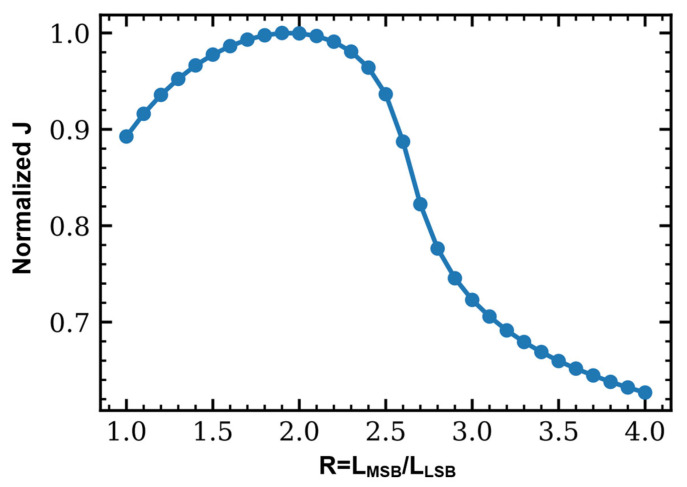
Normalized trade-off objective J  versus segment-length ratio.

To clarify the rationale of selecting the segment-length ratio, we performed a ratio sweep under a fixed total phase-shifter length budget of $L_{tot}=L_{long}+L_{short}$ and fixed dual-path driver settings. In this architecture, the long segment mainly contributes to the low-frequency modulation efficiency and thus dominates the extinction-ratio ER and OMA retention, whereas the short segment is dedicated to the high-frequency correction enabled by the peaking path. To quantitatively capture the trade-off between bandwidth extension and ER retention, we define a compact objective function:

(3) $$J\left(r\right)=BW_{3dB}\left(R\right)\cdot ER_{rel}\left(R\right)$$

where $R=L_{MSB}/L_{LSB}$, $BW_{3dB}$ is the EO 3 dB bandwidth referenced to the low-frequency magnitude ∣H(0)∣, and $ER_{rel}$ is a normalized low-frequency modulation-depth metric derived from ∣H(0)∣ and used as ER and OMA retention proxy under the same operating point. As shown in Figure 2, J(R) exhibits a clear maximum around R≈2, indicating that a near 2:1 partition provides the best overall balance within the explored range. When R<2, the long segment becomes too short and the low-frequency modulation efficiency decreases, leading to a degraded ER/OMA margin. Conversely, when R>2, the long segment dominates the junction capacitance and RC loading, which reduces the effective electrical bandwidth and weakens the benefit of high-frequency correction. Therefore, we adopt $L_{MSB}:L_{LSB}=2:1$ as a practical and near-optimal choice for jointly achieving bandwidth extension while maintaining ER and OMA.

**Figure 3:**
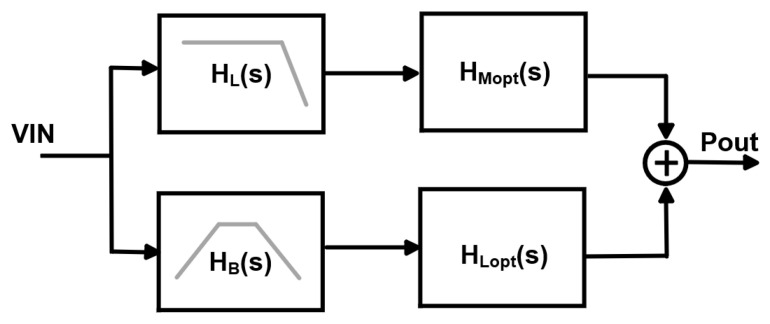
Dual-segment microring and frequency-division co-transmission architecture.

As shown in Figure 3, the proposed feedforward dual-segment MRM driver can be modeled by an equivalent two-path system. The input electrical signal $V_{\mathrm{IN}}(s)$ is applied to two parallel driving paths. The first path is an electrical low-pass filter $H_{L}(s)$, whose transfer function is approximated by a first- or higher-order low-pass response that covers the low- and mid-frequency components from 0 to $f_{N}$. The second path is a band-pass network $H_{B}(s)$, which is used to provide controlled gain near the Nyquist frequency. After passing through these two electrical networks, the resulting signals drive the long and short phase segments of the microring, respectively. The corresponding electro-optical transfer functions of these two segments are denoted as $H_{\mathrm{Mopt}}(s)$ and $H_{\mathrm{Lopt}}(s)$, where the former represents the MSB (long) segment and the latter represents the LSB (short) segment. Under small-signal conditions, their optical responses are linearly superposed at the output. The overall electro-optical transfer function of the proposed feedforward architecture is therefore

(4) $$H_{\mathrm{EO},\mathrm{dual}}\left(s\right)=H_{L}\left(s\right)H_{\mathrm{Mopt}}\left(s\right)+H_{B}\left(s\right)H_{\mathrm{Lopt}}\left(s\right)$$

when the phases of the two paths are approximately aligned over the target bandwidth, this expression can be interpreted as follows. The low-pass path provides the dominant baseband gain, while the high-frequency path supplies a shaped peaking profile around the Nyquist frequency. This yields a larger electro-optical 3 dB bandwidth and a peaked response at the Nyquist frequency with almost no degradation in the extinction ratio.

Furthermore, the low-pass path $H_{L}(s)$ should maintain a maximally flat amplitude response up to the Nyquist frequency and provide the main gain at DC and low frequencies. For the high-frequency path $H_{B}(s)$, the peaking frequency $f_{\mathrm{peak}}$ should be placed slightly higher than the target Nyquist frequency (e.g., $f_{\mathrm{peak}}\approx 1.2f_{N}$). In this way, the high-frequency path peaks the electro-optical response in the mid-to-high frequency region where the long-segment MRM response starts to roll off. The quality factor of the peaking profile should not be too high, preventing a sharp resonance with strong group-delay variation around $f_{\mathrm{peak}}$, which would cause ringing and overshoot in the time domain. The gain of the short segment must be carefully controlled to align with the long-segment gain. The low-pass path should dominate at DC and low frequencies so that the peaking path is effectively negligible in this region and only contributes at high frequencies.

**Figure 4:**
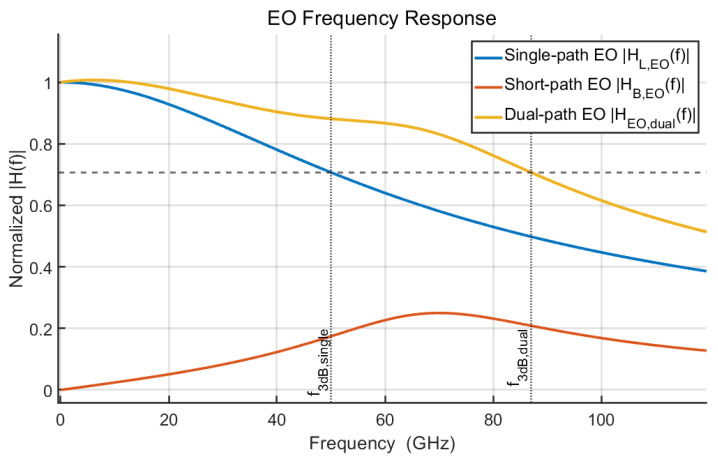
Simulated bandwidth extension with the proposed dual-segment microring driving scheme.

The bandwidth extension of the dual-path driver is evaluated by simulation. For the reference case, the single-path driver response is modeled as an ideal low pass, giving an overall electro-optical 3 dB bandwidth of about 50 GHz. When a short-segment peaking path is added with a center frequency of 70 GHz, quality factor Q=1.5, and relative gain coefficient $a_{2}=0.25,$ the gain is peaked in the 60–90 GHz range. The effective 3 dB bandwidth is extended from 50 GHz to 87 GHz, as shown in Figure 4, demonstrating substantial bandwidth enhancement.

### 2.3. Bandwidth Benefit of Segmented Microring Modulation

To quantitatively assess the advantage of the dual-segment feedforward architecture over a conventional single-segment microring, we analyze the equivalent small-signal transfer characteristics of the link. For a single-segment MRM, the small-signal electro-optical transfer function can be expressed as

(5) $$H_{EO,single}\left(f\right)=H_{opt}\left(f;Q\right)\cdot H_{elec}\left(f\right),$$

where $H_{opt}(f;Q)$ is the Lorentzian optical response determined by the cavity quality factor Q, and $H_{elec}(f)$ is the electrical transfer function determined jointly by the driver stage, package, and load. In most practical designs, $H_{elec}(f)$ can be approximated as a second-order low-pass response with electrical 3 dB bandwidth $f_{3,elec}$. These two responses together define the overall electro-optical bandwidth of the single-segment link.

**Figure 5:**
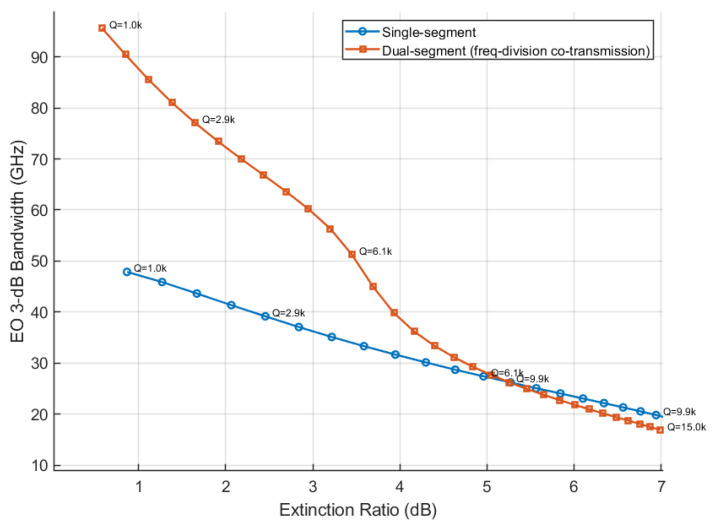
Bandwidth improvement of segmented microring modulation.

At a wavelength of 1310 nm, a 2-$V_{\mathrm{pp}}$ drive swing, and a bias set at the −3 dB operating point, we perform equivalent electro-optical simulations for both single- and dual-segment microring structures, as shown in Figure 5. For the electrical single-segment baseline, the 3 dB bandwidth is set to 50 GHz. In the dual-segment case, the long segment has an electrical bandwidth of 45 GHz, and the short segment is driven by a narrowband peaking network with a center frequency of 60 GHz and Q=1.5.

The results show that in the low-ER/low-Q region, the overall link is mainly limited by the electrical bandwidth. At the same extinction ratio, the dual-segment modulation and driving scheme may effectively extend the electro-optic bandwidth. In the high-ER/high-Q region, the link becomes limited by the optical bandwidth. Because the modulation efficiency of the dual-segment structure is reduced in this case, it does not provide additional bandwidth benefit at the same extinction ratio. In a 200 Gb/s microring-based transmitter, the dual-segmented MRM achieves a higher effective electro-optic bandwidth than the single-segmented MRM when the extinction ratio is constrained to <4 dB.

## 3. Circuit Implementation

### 3.1. System-Level Architecture

**Figure 6:**
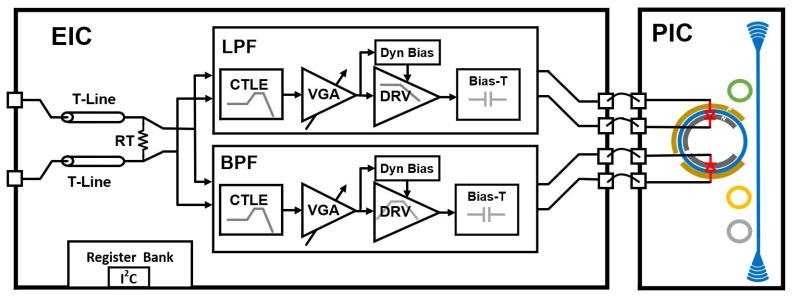
System-level architecture.

This work targets a 100-GBaud dense WDM (DWDM) microring transmitter and adopts a feedforward scheme with a dual-segment MRM. The overall system architecture is shown in Figure 6. The transmitter consists of an electrical integrated circuit (EIC) implementing the dual-path driver and a photonic integrated circuit (PIC) implementing the dual-segment microring modulator.

High-speed differential signals are delivered between chips through on-chip transmission lines using die-to-die (D2D) interconnections. Proper termination ensures impedance matching at the dual-path input. The differential input data is broadcast to both driver paths in parallel through the input distribution network. The two paths are intentionally designed with complementary frequency-selective transfer functions, so that the LF content is predominantly carried by the broadband LF (MSB) path, whereas the HF correction is mainly provided by the narrowband HF (LSB) path via a peaking response around the target high-frequency region (e.g., near the Nyquist frequency). The LF-path output drives the long segment to preserve modulation efficiency and ER contribution, while the HF-path output drives the short segment to supply high-frequency boost. The two segment contributions are then superposed on the same microring resonant mode, enabling optical domain recombination without an electrical combiner and its associated parasitics.

Both paths adopt a CTLE–VGA–DRV–Bias-T topology. The upper path (MSB path) is a broadband low-frequency path that drives the long segment (MSB). The lower path (LSB path) is a narrowband high-frequency path that drives the short segment (LSB). In both paths, the CTLE compensates for the high-frequency loss from the D2D interconnection and package, and its effective response can be approximated as a first-order low-pass with peaked high-frequency gain. The VGA provides 5–8 dB of adjustable gain to equalize the overall gain between the two paths. In the MSB path, the DRV is a high-swing, wide-bandwidth differential output stage that provides more than 2-$V_{\mathrm{ppd}}$ to the MRM load. Its electrical −3 dB bandwidth is designed to be 45–50 GHz so that it can efficiently drive the main modulation segment. In the LSB path, the DRV employs the LC-resonant structure whose resonant frequency is set to around 60 GHz. This stage provides an adjustable peaking profile at the Nyquist region frequency to compensate for the high-frequency components of the signal. A Bias-T is placed at the output of each path. It AC-couples the DRV to the MRM electrode and blocks DC at the driver. The DC reverse bias is injected through a high-impedance bias branch. The two Bias-Ts share the same DC bias rail. Thus, both MRM segments have the same DC operating point, while the RF modulation remains path specific.

### 3.2. MSB Path Driver Output Stage

**Figure 7:**
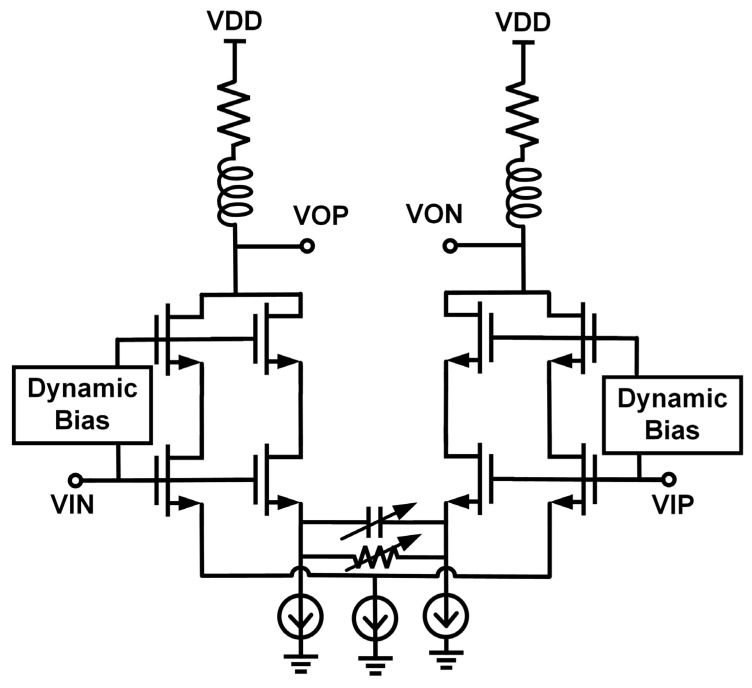
MSB path driver output stage.

The MSB path represents the low-pass branch $H_{L}(s)$ in the feedforward architecture. For 100-GBaud PAM4 signaling, this stage must deliver a 2-$V_{\mathrm{ppd}}$ differential swing into the long-segment MRM’s capacitive load, while providing an electrical 3 dB bandwidth of about 50 GHz. Most of the baseband and mid-frequency components from 50 GHz are modulated by the long segment, which relaxes the linearity and power requirements of the high-frequency compensation path. As shown in Figure 7, the long-segment driver adopts a current-mode output stage. To overcome bandwidth limitations, a 4-bit resistor array and a 4-bit capacitor array are used for source degeneration, together with a shunt-peaking inductor to extend the high-frequency response. Shunt resistors are inserted between the differential outputs to reduce the effective output impedance in the differential mode and improve bandwidth by reducing the output RC time constant. A dynamic biasing network is introduced to prevent device breakdown at large output swing and to maintain good linearity, as described in Section 3.4.

### 3.3. LSB Path Driver Output Stage

**Figure 8:**
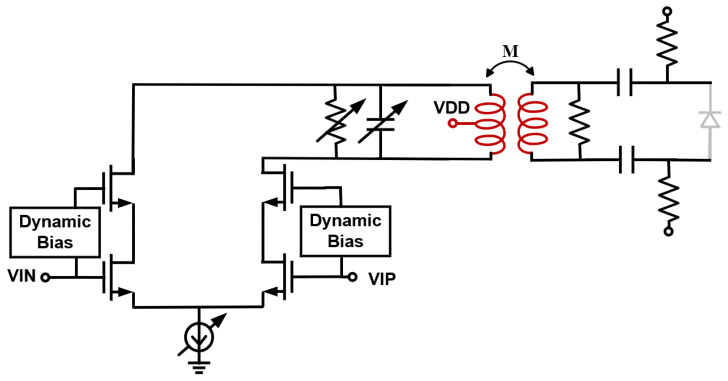
LSB path driver output stage with a double-tuned transformer.

The LSB path implements the feedforward branch for high-frequency peaking profile $H_{B}(s)$. It provides additional gain around the Nyquist frequency to complement the high-frequency roll-off of the MSB path. For 100-GBaud PAM4 signaling, the target center frequency $f_{\mathrm{peak}}$ is chosen around $(1.1\;-\;1.3)\;f_{N}$, with an effective quality factor $Q_{\mathrm{BPF}}$ of 1–4 and a gain of 30–50% of the MSB path, so that the combined electro-optical response remains flat near $f_{N}$ and the overall 3 dB bandwidth reaches about 70 GHz. As shown in Figure 8, the short-segment driver uses current-mode topology with a double-tuned transformer for in-band peaking control. The transformer employs two inductors with identical inductance and a controlled coupling coefficient M, which enhances the narrowband gain around the resonance. The active RLC network is tuned by 4-bit Rdac and 4-bit Cdac arrays, enabling independent adjustment of the resonant frequency and effective Q-factor. The secondary winding also uses Rdac to tune the coupling strength and avoid an underdamped double-tuned behavior. Because the required output swing on the LSB segment is smaller than that of the MSB path, this stage can operate with a lower bias current and reduced power. In addition, the Rdac/Cdac networks and the coupling coefficient k help absorb routing, ESD, and package parasitics, making it easier to realize a high-frequency, low-Q peaking driver.

### 3.4. Dynamic Bias Network

To prevent stacked transistors being overdriven at high output swing, a dynamic bias network is employed. This circuit also keeps the cascode devices in saturation and improves the linearity for PAM4 signals. Since the dynamic bias is applied to common-gate devices, it has negligible impact on the small-signal gain and bandwidth of the main driver.

**Figure 9:**
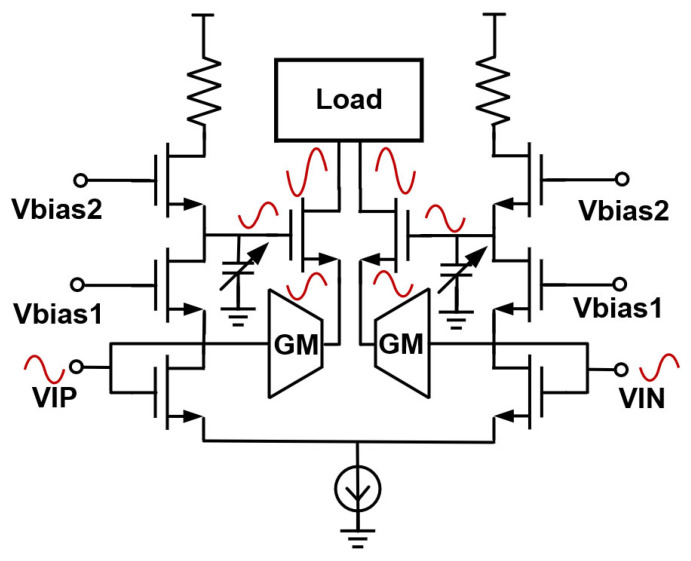
Implementation of the dynamic bias circuit.

The circuit is shown in Figure 9. The core of the dynamic bias consists of a fully differential pair that drives stacked common-gate devices. The main amplification path comprises the GM stage, the cascode devices, and the load, while the dynamic bias branch uses a scaled-down replica of the main devices with a bias current of about one tenth of the main driver. The DC level of the dynamic bias is set by a 4-bit voltage DAC, and the gain of the dynamic bias path is about one quarter to one third of the main stage. This configuration ensures that, even with a 2-$V_{\mathrm{ppd}}$ output swing, the voltage differences across the cascode devices in the main path remain below 0.9 V, thereby avoiding voltage breakdown.

## 4. System-Level Simulation

### 4.1. Peaking Frequency and Group-Delay Constraints

In the feedforward dual-segment architecture, the peaking frequency $f_{\mathrm{peak}}$ and quality factor $Q_{\mathrm{short}}$ of the LSB path are the key design parameters. For a 100 GBaud PAM4 (200 Gb/s) link, the target electro-optical 3 dB bandwidth in this work is set to at least 70 GHz. Taking packaging parasitics into account, the MSB path is modeled as a first-order low-pass with an electro-optical 3 dB bandwidth of 50 GHz, which provides the dominant in-band gain from DC to $f_{N}$. The MSB path carries the dominant modulation weight and thus is designed to preserve the in-band signal integrity up to the Nyquist frequency $f_{N}$. Placing the MSB path corner close to $f_{N}$ minimizes the required high-frequency correction delegated to the LSB path, thereby reducing the necessary peaking strength and the associated group-delay ripple and time-domain ringing. The LSB path is represented by a second-order band-pass network with center frequency $f_{\mathrm{peak}}$ and quality factor $Q_{\mathrm{short}}$. Bandwidth extension and group-delay variation are jointly considered to evaluate the impact of $f_{\mathrm{peak}}$ and $Q_{\mathrm{short}}$ on the dual-path feedforward scheme. Two design constraints are adopted:

1. Bandwidth constraint:

(6) $$f_{3\mathrm{dB},\mathrm{dual}}\geq 70\;\mathrm{GHz}\approx 1.3–1.4\times f_{3\mathrm{dB},\mathrm{long}}$$

2. Group-delay constraint:

(7) $$\Delta \tau ≜\underset{f\in \left[0,f_{N}\right]}{\max}\tau_{g}\left(f\right)|-|\underset{f\in \left[0,f_{N}\right]}{\min}\tau_{g}\left(f\right).$$

**Figure 10:**
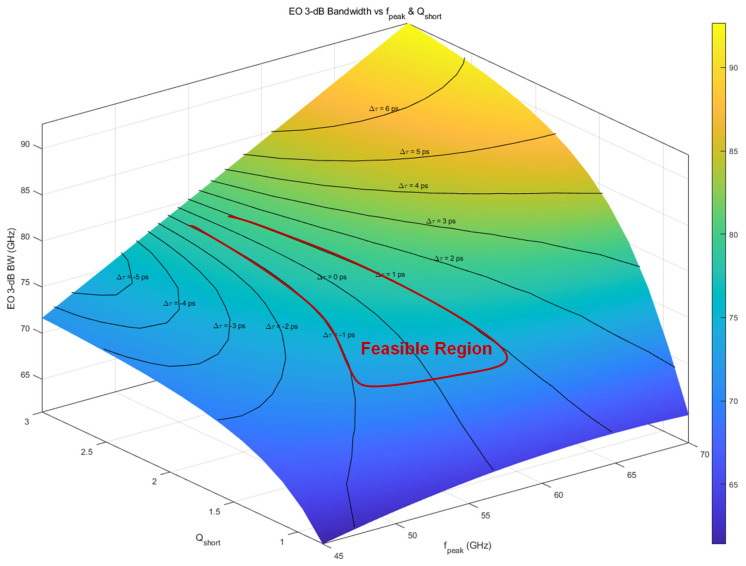
Effect of peaking frequency and Q factor on bandwidth.

Figure 10 plots the electro-optical 3 dB bandwidth as a function of $f_{\mathrm{peak}}$ and $Q_{\mathrm{short}}$, with contour lines indicating ∣Δτ∣. The results show that when $f_{\mathrm{peak}}\approx 1.1–1.3f_{N}$ and $Q_{\mathrm{short}}\approx 1.5–2.0$, the overall 3 dB bandwidth can be extended from 50 GHz (MSB path only) to 80–90 GHz, while ∣Δτ∣ remains within approximately 1 ps. This region is therefore identified as the preferred design window for the short-segment peaking path.

### 4.2. Dual-Segment Modulator Gain Matching

The equivalent model in Section 4.1 identifies an optimal region of $\left(f_{peak},Q_{short}\right)$ for the short segment to compensate the long-segment bandwidth limit and the optical Q-limited response. The remaining question is practical: whether the implemented LSB driver provides two nearly orthogonal tuning knobs that can (i) place the peaking center frequency and (ii) shape the damping/peaking strength in a controlled manner. To answer this, circuit-level AC simulations are performed on the LC-peaking network in the high-frequency path.

#### 4.2.1. Orthogonal Tuning Knobs: Frequency Placement vs. Damping Control

I. Capacitor array mainly controls $f_{peak}$.

**Figure 11:**
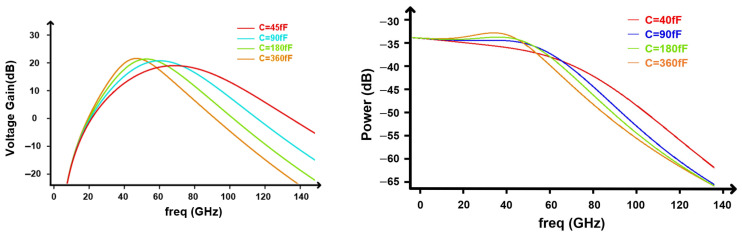
Tuning of the narrowband resonant frequency.

Figure 11 characterizes the capacitor array in the transformer output network. Varying the capacitance shifts the resonant peaking frequency over a wide range, while keeping the peak level nearly constant. As the capacitance increases from 45 fF to 360 fF, $f_{peak}$ moves monotonically from about 70 GHz down to 40 GHz, whereas the peak voltage gain remains within 28–30 dB (left plot). The corresponding output power response (right plot) exhibits the same resonant shift. A smaller capacitance pushes the resonance to a higher frequency and retains higher high-frequency power, while a larger capacitance moves the resonance down and accelerates the roll-off due to heavier capacitive loading. These results establish a monotonic mapping from the capacitor code to $f_{peak}$, indicating that the capacitor array serves as a robust knob to place the peaking frequency.

II. Shunt resistor array mainly controls $Q_{short}$ and peak magnitude.

**Figure 12:**
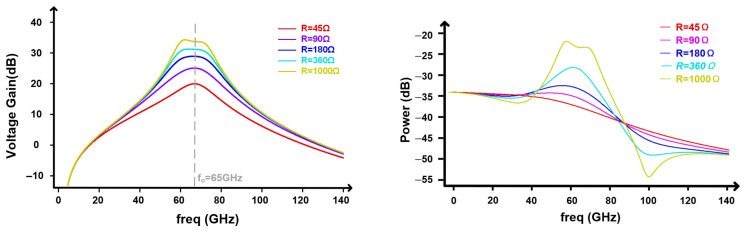
Gain response of the peaking network for different shunt resistances.

Figure 12 characterizes the shunt resistor array as a means to control the damping and peaking strength of the transformer response. With $f_{peak}\approx 65\;GHz$, increasing the shunt resistance from 45 Ω to 1 kΩ transitions the response from overdamped toward a near-critically damped condition. The voltage gain response shows progressively stronger peaking and a higher effective Q (left plot). The output power response confirms the same trend: the in-band power peak around $f_{peak}$ increases, while the out-of-band response becomes less flat, including a deeper dip at higher frequency (right plot). This result highlights a clear trade-off between peaking strength and high-frequency power flatness; accordingly, the resistor code should be chosen to provide sufficient compensation without driving the network into an underdamped regime that would induce time-domain ringing.

#### 4.2.2. Tuning Procedure and Design Criteria

Based on the above mapping, gain matching between the two segments is performed using a separable two-step procedure.

Step 1 (frequency alignment): Select the capacitor code such that $f_{peak}$ is placed near the target region predicted by Section 4.1 (typically $f_{peak}\approx 1.1–1.3f_{N}$), so that the LSB peaking overlaps with the MSB roll-off near the band edge. The selection criterion is to minimize the residual gain error around $f_{N}$.

Step 2 (shape and stability): With the capacitor code fixed, adjust the resistor code to set $Q_{short}$ and peaking magnitude. The resistor is increased until the in-band ripple meets the target, and then reduced if an excessive out-of-band dip appears or if time-domain ringing is observed in transient simulations. In practice, the resistor code is chosen to approach a near critically damped response to balance compensation strength and stability.

Overall, the LSB driver provides a sufficient tuning margin to realize $f_{peak}\approx \left(1.1–1.3\right)f_{N}$ and $Q_{short}\approx 1.5–2.0$, consistent with the theoretical design space in Section 4.1. The capacitor array enables reliable frequency placement, while the resistor array provides controlled damping and peaking strength adjustment, thereby supporting systematic gain matching between the dual segments.

### 4.3. Frequency Response and Transient Response

**Figure 13:**
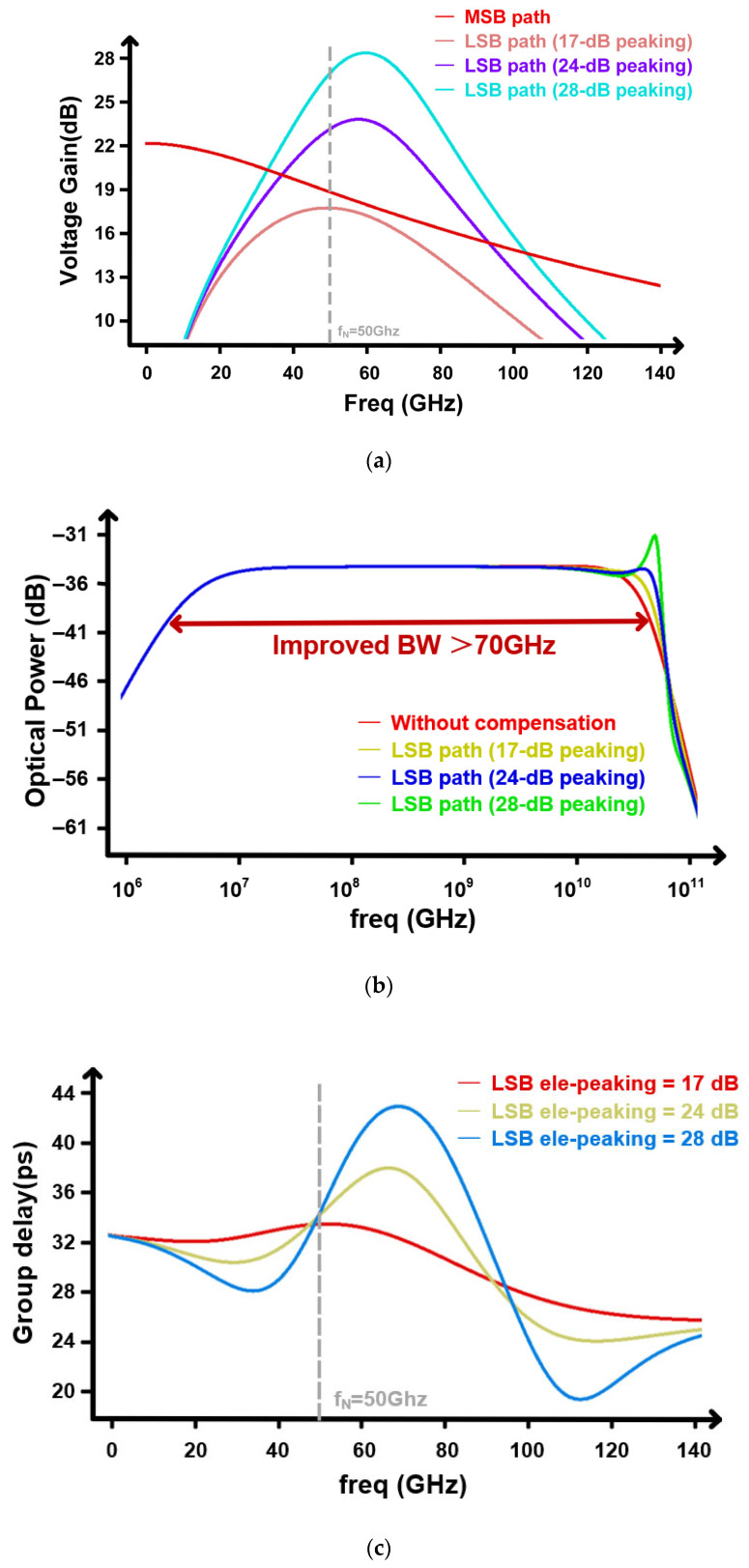
(**a**) Dual-path electrical frequency response. (**b**) Electro-optical frequency response. (**c**) Electro-optical group delay.

With the design window for $f_{\mathrm{peak}}$ and $Q_{\mathrm{short}}$ determined, we next evaluate the full electro-optical frequency response of the transmitter link. AC simulations are carried out for the complete chain including the EIC, the dual-segment MRM modeled in Verilog-A, and the package network, using Spectre as the circuit simulator. Figure 13 summarizes the frequency-domain behaviors of the proposed dual-path transmitter.

Figure 13a plots the electrical voltage gain responses of the two paths at the MRM load. The MSB path exhibits a broadband low-pass characteristic, providing the dominant low-to-mid-frequency drive for the long segment. In contrast, the LSB path is configured as a narrowband high-frequency boosting path with an LC-resonant output network, where the electrical peaking around the Nyquist region is programmable. By adjusting the LSB electrical peaking level (from 17 dB to 28 dB), the high-frequency contribution of the LSB path can be increased to compensate for the roll-off of the MSB path, thereby shaping the overall stitching profile near $f_{N}=50$ GHz.

Figure 13b plots the electro-optical (EO) small-signal response of the transmitter for four configurations: without compensation and with the LSB path enabled under three electrical peaking settings (17/24/28 dB). Without compensation, the EO response exhibits an earlier high-frequency roll-off, limiting the effective EO bandwidth. When the LSB path is activated, the high-frequency components are reinforced and the EO response is substantially flattened near the band edge, resulting in an improved −3 dB bandwidth beyond 70 GHz, as highlighted in the figure. As the LSB peaking level increases from 17 dB to 24 dB and 28 dB, the bandwidth extension becomes more pronounced, but excessive peaking (e.g., 28 dB) introduces a stronger high-frequency gain boost near the roll-off region, which typically correlates with larger group-delay ripple and may degrade the time-domain eye performance. The nominal setting therefore provides a desirable compromise between bandwidth extension and group-delay flatness.

Figure 13c further plots the corresponding EO group-delay responses, highlighting the bandwidth–dispersion trade-off introduced by the programmable peaking. As the LSB electrical peaking is increased, the group-delay ripple becomes more pronounced, especially around the peaking region, which may translate into larger timing distortion and potential eye degradation in the time domain. Therefore, the nominal setting provides a desirable compromise between EO bandwidth extension and group-delay flatness, while avoiding excessive resonant peaking.

**Figure 14:**
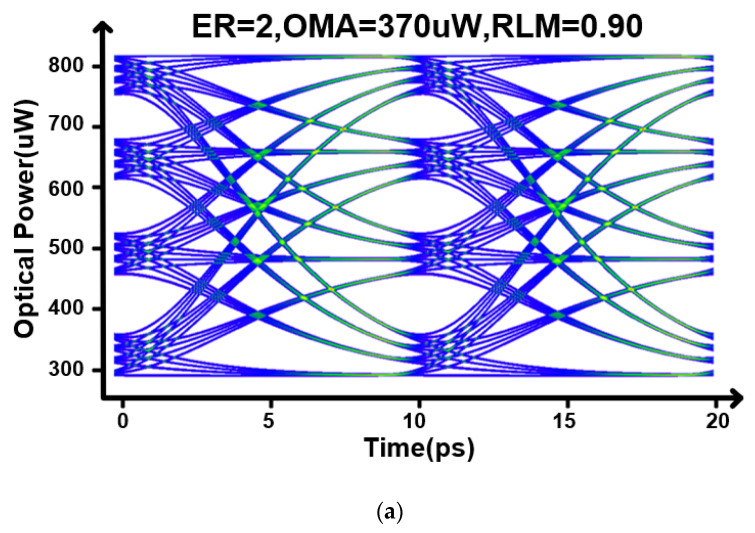

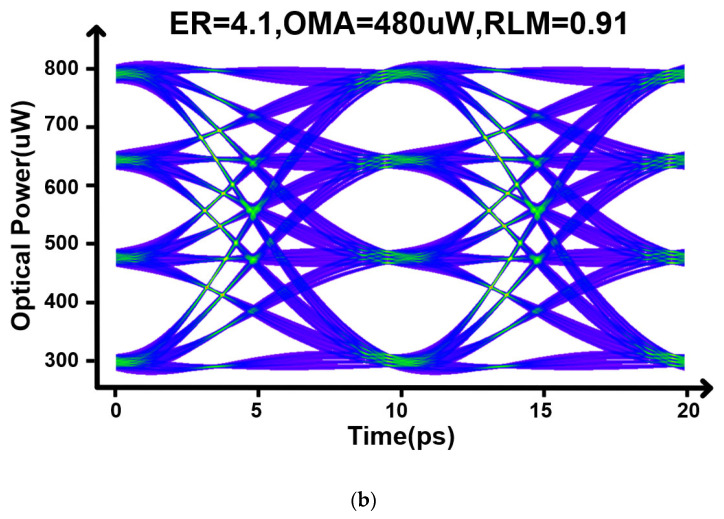
(**a**) Eye diagram without compensation. (**b**) Eye diagram with compensation.

Figure 14 compares the 200 Gb/s PAM4 optical eye diagrams under different compensation settings. In Figure 14a, only the MSB path is enabled and the transmitter behaves as a bandwidth-limited link. The insufficient high-frequency content leads to pronounced inter-symbol interference (ISI). As a result, the extinction ratio (ER) is limited to 2 dB with an RLM of 0.90, indicating a non-negligible penalty from the constrained electro-optic bandwidth. In Figure 14b, the LSB path is activated with $f_{\mathrm{peak}}\approx 65$ GHz to provide high-frequency boosting near the Nyquist region and to smooth the frequency-domain stitching between the two paths. In the time domain, the PAM4 transitions become sharper and the ISI is effectively reduced. Quantitatively, the ER improves from 2 dB to 4.1 dB, and the OMA increases from 370 μW to 480 μW, confirming that the proposed dual-path compensation enhances the usable eye opening without sacrificing modulation amplitude.

**Figure 15:**
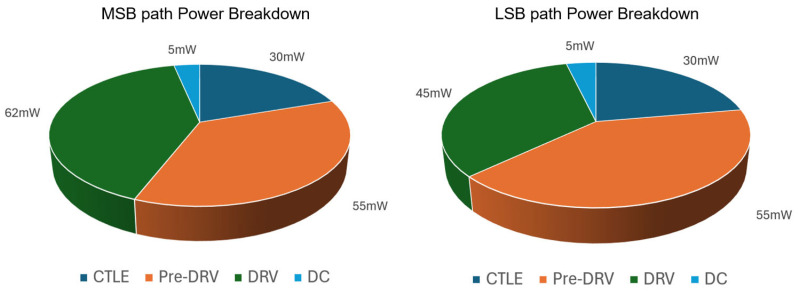
Power breakdown of TX.

The performance of the proposed transmitter is summarized in Table 1 and compared with representative state-of-the-art microring-based designs. Enabled by the co-design of a dual-segment microring modulator and a two-path feedforward driver, this work achieves 200 Gb/s PAM4 operation in 28 nm CMOS with a segmented microring. With the high-frequency compensation enabled, the link-level electro-optical 3 dB bandwidth is extended from ~50 GHz to ~75 GHz, leading to a clear improvement in the simulated PAM4 optical eye. This work also maintains competitive energy efficiency (1.5 pJ/bit), as shown in Figure 15, while providing a practical TX extinction ratio of 4.1 dB. Overall, Table 1 highlights a favorable speed–efficiency–ER trade-off achieved by systematic bandwidth shaping.

### 4.4. Practical Robustness Considerations

In practical implementation and volume production, the robustness of the proposed dual-path segmented MRM transmitter is mainly limited by three non-idealities: (i) the matching accuracy between the LF and HF paths (e.g., relative gain/peaking strength and effective peaking frequency), where mismatch can cause under-/over-compensation and degrade PAM4 eye metrics such as ER/OMA and level uniformity (RLM); (ii) the level of cross-segment RF coupling between the long and short segments—as the operating speed increases and the segments are placed in close proximity, parasitic pad/interconnect coupling may become non-negligible, raising the question of how much RF leakage occurs across segments; such unintended injection can disturb the intended LF/HF signal partitioning, introduce additional waveform distortion, and potentially lead to eye closure; and (iii) PVT variations, which shift device parameters and operating conditions and thereby perturb the effective HF-path response and the resulting modulation quality.

To quantify the level of cross-segment RF coupling between the MSB and LSB segments placed in close proximity, we define the voltage isolation (MSB as aggressor and LSB as victim) as

(8) $$I_{MSB\to LSB}\left(f\right)=20{log}_{10}|\frac{V_{LSB}\left(f\right)}{V_{MSB}\left(f\right)}|\;(\mathrm{dB})$$

where $V_{MSB}(f)$ is the small-signal pad voltage on the MSB segment and $V_{LSB}(f)$ is the induced pad voltage on the LSB segment (more negative $I_{MSB\to LSB}(f)$ indicates better isolation; a similar trend is observed for $I_{LSB\to MSB}(f)$). The inter-segment coupling capacitance $C_{c}$ is obtained from parasitic/EM simulation of the segment pads/interconnects, and the extracted nominal value is approximately $C_{c}\approx 8\;fF$, which is on the same order as the LSB junction capacitance $C_{jLSB}$ in this design.

**Figure 16:**
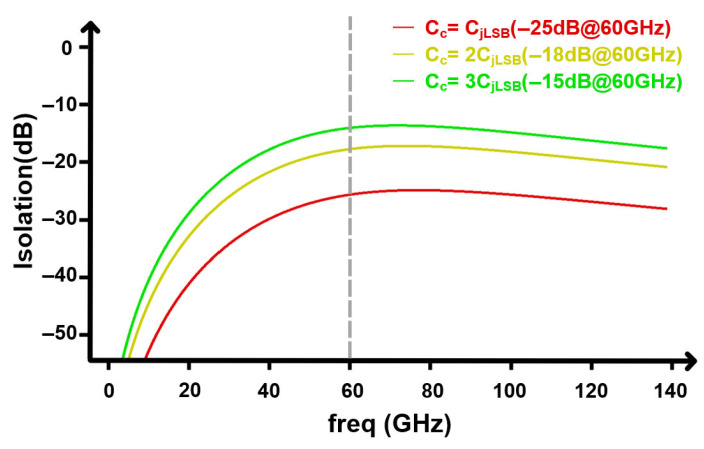
Simulated voltage isolation versus frequency under swept inter-segment coupling capacitance $C_{c}$.

**Figure 17:**
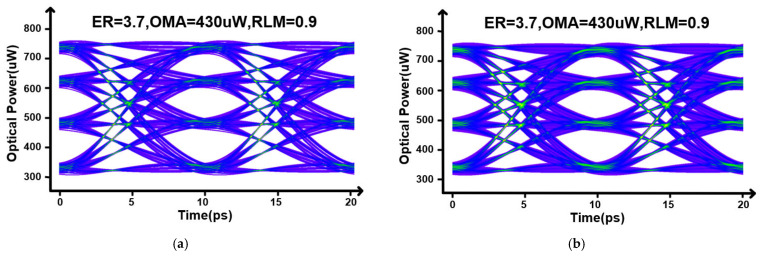
Optical PAM4 eye diagrams under cross-segment RF coupling conditions. (**a**) Without cross-segment RF coupling. (**b**) With cross-segment RF coupling.

Figure 16 plots $I_{MSB\to LSB}(f)$ versus frequency and further stresses the coupling by sweeping $C_{c}$ to emulate aggravated parasitic coupling at higher operating speeds. Around the HF peaking region (∼60 GHz), the simulated isolation at 60 GHz is is approximately −25 dB, −18 dB, and −15 dB for $C_{c}=1\times C_{j,LSB}$, $2\times C_{j,LSB}$, and $3\times C_{j,LSB}$, respectively. Corresponding time-domain optical PAM4 eye diagrams under these coupling conditions are shown in Figure 17, where only minor distortion is observed and no obvious eye closure occurs even under the $3\times C_{j,LSB}$ stressed coupling case, indicating tolerance to moderate cross-segment RF leakage.

Although the extracted cross-segment isolation is finite, its impact on the PAM4 eye is limited in this architecture. This is because the LF- and HF-path waveforms are derived from the same input, so any leakage coupled between the MSB and LSB segments is largely correlated and phase-aligned rather than an independent aggressor. In addition, the two segments primarily operate over different effective frequency ranges, so the coupled component overlaps only partially with the victim segment’s dominant content and therefore does not strongly disturb the intended signal partitioning. Consistent with this intuition, the optical PAM4 eye diagrams show negligible degradation when enabling the extracted coupling.

To assess robustness against process and temperature variations, we evaluate the transmitter behavior under representative corners while introducing a controlled ±10% variation (α) of the HF-path peaking frequency to capture potential corner-induced drift and modeling uncertainty of the high-frequency boosting network. Table 2 summarizes the key optical PAM4 metrics under the nominal setting and the stressed settings, where the stressed cases are chosen to represent the most unfavorable peaking-frequency shift within each corner.

**Figure 18:**
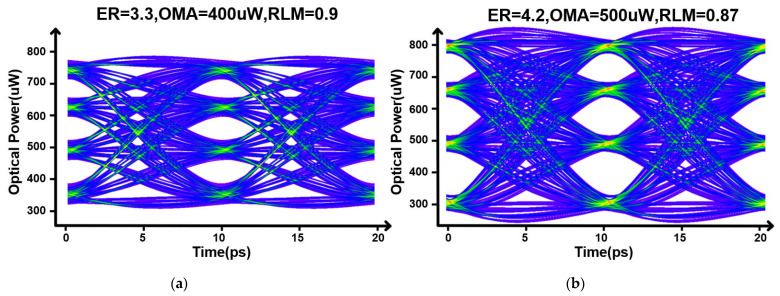
Representative optical PAM4 eye diagrams under different corners with HF-path peaking-frequency variation. (**a**) SS corner with a −10% peaking-frequency shift. (**b**) FF corner with a +10% peaking-frequency shift.

Figure 18 provides representative optical PAM4 eye diagrams for the stressed SS and FF conditions. Under the SS corner with a −10% peaking-frequency shift ($f_{0}=0.9f_{0,nom}$), the eye remains open with ER = 3.3 dB, OMA = 400 µW, and RLM = 0.9. Under the FF corner with a +10% peaking-frequency shift ($f_{0}=1.1f_{0,nom}$), the eye also remains open with ER = 4.0 dB, OMA = 400 µW, and RLM = 0.9. These results indicate that, even when PVT corners are combined with moderate peaking-frequency drift, the proposed architecture maintains a clear PAM4 eye opening and preserves level uniformity without introducing noticeable eye closure.

## 5. Conclusions and Discussion

This work addresses the bandwidth–extinction-ratio trade-off of high-Q microring modulators in DWDM transmitters and proposes a feedforward scheme for dual-segment MRMs. By constructing a high-swing low-pass path for the long segment and a high-gain narrowband path for the short segment of the MRM, the two electrical signals are recombined in the optical domain at the same resonance. As a result, effective electro-optical bandwidth extension is achieved with only a modest penalty in extinction ratio. The main contributions are summarized as follows.

1. ER–Q–bandwidth constraint model for single-segment MRMs.
  An analytical ER–Q–bandwidth model is derived from the Lorentzian optical response, correlating extinction ratio, cavity quality factor, and optical 3 dB bandwidth. This model clarifies the inherent conflict between high Q, high ER, and large bandwidth, and motivates the use of segmented modulation. It also quantifies the achievable bandwidth gain at a fixed ER when moving from single-segment to dual-segment operation.
2. Feedforward dual-segment architecture and its design space.
  A dual-path, dual-segment driving architecture is proposed, in which the input signal is decomposed into low- and high-frequency components that drive the long and short MRM segments, respectively, and are superposed in the same cavity. By formulating the combined electro-optical transfer function and sweeping the peaking frequency $f_{\mathrm{peak}}$ and quality factor $Q_{\mathrm{short}}$ for the LSB path, we identify an optimal region where ∣Δτ∣≤1 ps and the electro-optical 3 dB bandwidth is extended from 55 GHz to 80–90 GHz, corresponding to $f_{\mathrm{peak}}\approx 1.1–1.3{\;f}_{N}$ and $Q_{\mathrm{short}}\approx 1.5–2.0$.
3. Dual-path driver implementation and system-level validation.
  A complete dual-path driver is implemented, using a broadband CTLE–VGA–DRV–Bias-T chain in the MSB path to provide the in-band gain, and a programmable RC-peaking network in the LSB path to independently tune $f_{\mathrm{peak}}$ and the peak gain. Co-designed driver and MRM parameters yield about 1.5× electro-optical bandwidth extension and significantly improved 200 Gb/s PAM4 eye quality in system-level simulations.

Future work can proceed in three directions. First, closed-loop calibration of the low- and high-frequency path gains and automatic tuning of the peaking frequency/strength will be explored to stabilize the compensation performance against PVT variation and packaging/interconnect parasitics; a practical approach is to use measured EO frequency response and/or PAM4 eye metrics as feedback to select the optimal peaking code and LF/HF weighting with a lightweight search strategy. Second, the scalability of the proposed architecture toward 256 Gb/s and beyond, as well as multi-wavelength DWDM operation, will be investigated to evaluate its potential for Tb/s-class transmitters, where the key challenges include channel-to-channel dispersion and thermal drift/crosstalk; preliminary solutions include per-channel programmable peaking/weighting together with coordinated thermal control to maintain resonance alignment and uniform bandwidth–ER trade-offs across lanes and wavelengths. Third, the feedforward co-design concept will be extended to Mach–Zehnder modulators and co-packaged optics platforms to assess its generality for broadband electro-optical co-design in integrated transmitters, with emphasis on mapping the dual-path compensation strategy to different modulator transfer functions and validating its robustness under realistic package-level parasitics and system constraints.

## Figures and Tables

**Table 1:** Performance summary and comparison.

Reference	JSSC [16]	OI [17]	BCICTS [10]	OFC [18]	This Work
Technology	45 nm SOI	65 nm CMOS	28 nm CMOS	28 nm CMOS	28 nm CMOS
Type of microring	single	segmented	segmented	single	segmented
Bandwidth enhancement	CTLE	Two-Segment Optical DAC	CTLE + FFE	CTLE	Feedforward DRV
Channel speed	50 Gb/s NRZ	40 Gb/s PAM4	64 Gb/s NRZ	128 Gb/s PAM4	200 Gb/s PAM4
Power efficiency (pJ/bit)	3.5	4.38	5.6	1.5	1.44
TX ER (dB)	6.9	N/R*	3.2	3.8	4.1

N/R*: Not reported.

**Table 2:** Performance summary under different process variations.

Corner	Temp (°C)	Variation (α)	ER (dB) Min–Max	OMA (mW) Min–Max	RLM Min–Max
TT	27	0.9–1.1	3.5–4.1	420–480	0.9–0.92
SS	120	0.9–1.1	3.3–3.8	400–430	0.89–0.91
FF	−40	0.9–1.1	3.7–4.2	450–500	0.87–0.9

## Data Availability

Data underlying the results presented in this paper are not publicly available at this time but may be obtained from the authors upon reasonable request.
